# Supplementary material for: rpoB, a promising marker for analyzing the diversity of bacterial communities by amplicon sequencing
Source: BMC Microbiol. 2019 Jul 29;19:171. doi: 10.1186/s12866-019-1546-z (PMC6664775; doi:10.1186/s12866-019-1546-z)
Supplement: Supplementary file 2 — Rarefaction curves obtained by Illumina-amplicon sequencing of rpoB (A) and 16S (B) markers in 15 mock community samples (five mock communities, three replicates per mock) and the four replicates of the Steinernema glaseri nematode sample. (PPTX 100 kb) [file 12866_2019_1546_MOESM2_ESM.pptx]

## Slide 1
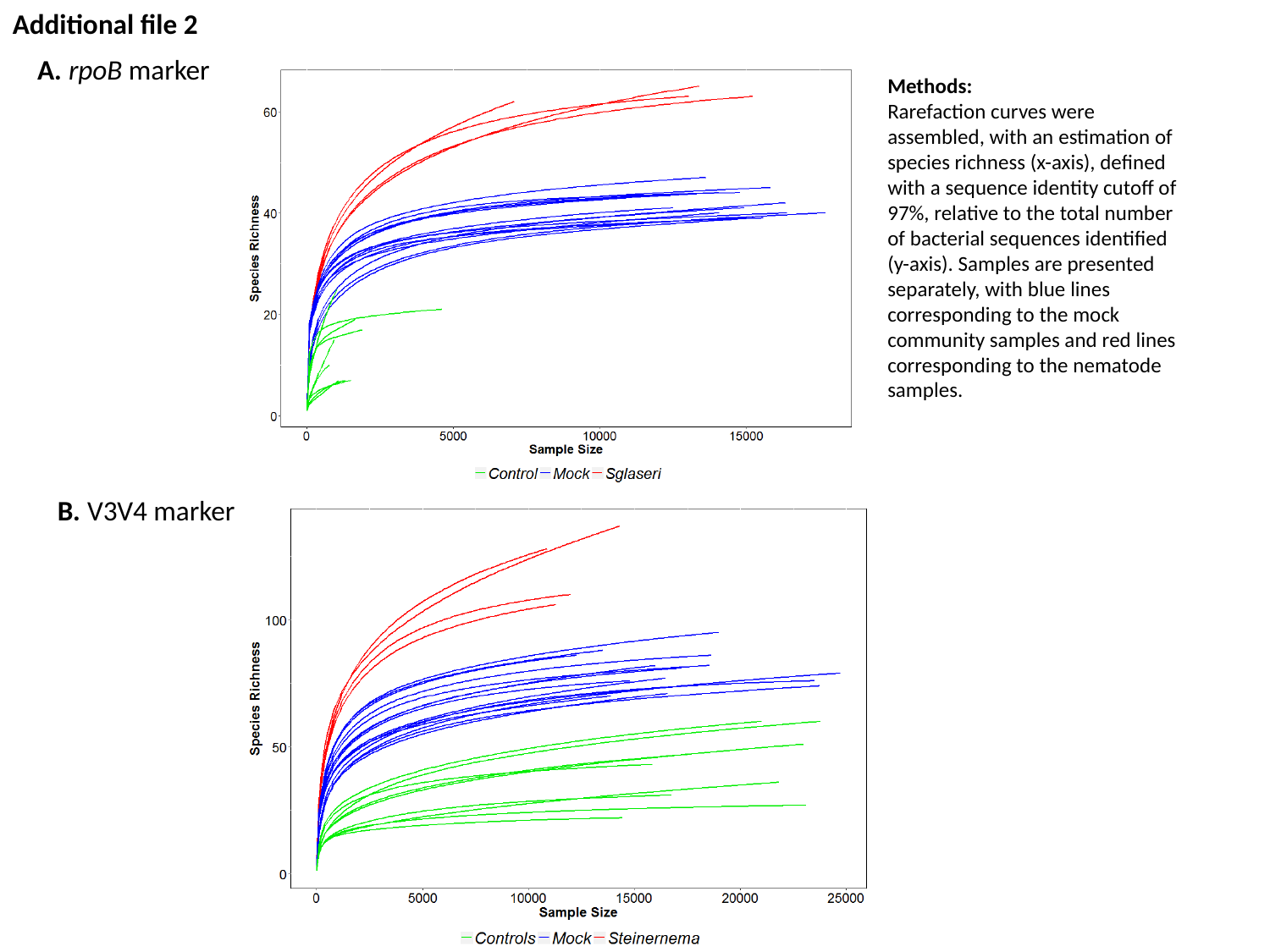

Additional file 2
A. rpoB marker
Methods:
Rarefaction curves were assembled, with an estimation of species richness (x-axis), defined with a sequence identity cutoff of 97%, relative to the total number of bacterial sequences identified (y-axis). Samples are presented separately, with blue lines corresponding to the mock community samples and red lines corresponding to the nematode samples.
B. V3V4 marker
