# Supplementary material for: rpoB, a promising marker for analyzing the diversity of bacterial communities by amplicon sequencing
Source: BMC Microbiol. 2019 Jul 29;19:171. doi: 10.1186/s12866-019-1546-z (PMC6664775; doi:10.1186/s12866-019-1546-z)
Supplement: Supplementary file 5 — Comparison of the observed and expected relative abundances of the bacterial communities obtained by Illumina-amplicon rpoB (A) and 16S (B) sequencing for the mock1, mock2, and mock3 communities. See Fig. 5 for Figure legend details. (PPTX 288 kb) [file 12866_2019_1546_MOESM5_ESM.pptx]

## Slide 1
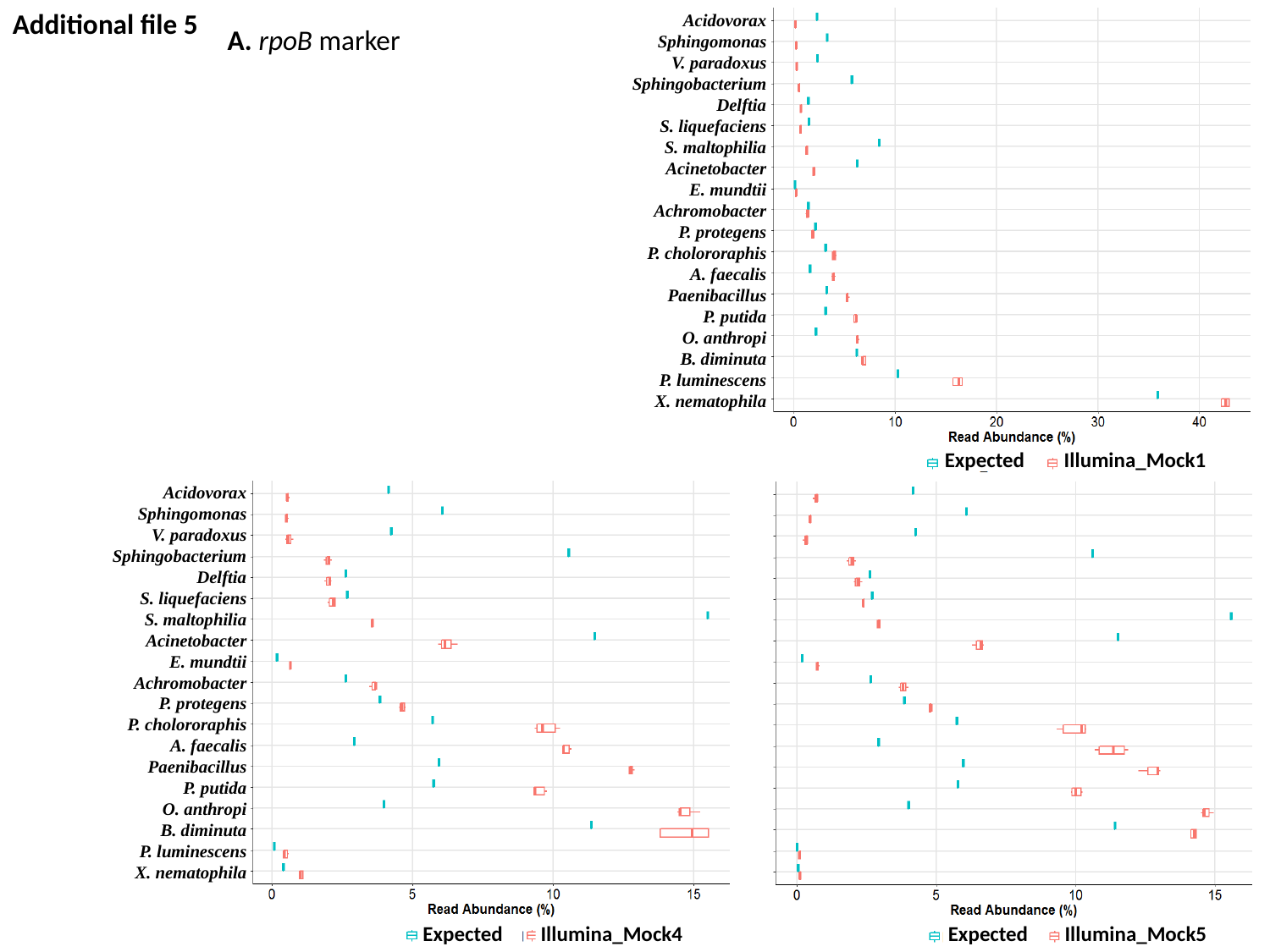

Additional file 5
Acidovorax
Sphingomonas
V. paradoxus
Sphingobacterium
Delftia
S. liquefaciens
S. maltophilia
Acinetobacter
E. mundtii
Achromobacter
P. protegens
P. cholororaphis
A. faecalis
Paenibacillus
P. putida
O. anthropi
B. diminuta
P. luminescens
X. nematophila
Expected
Illumina_Mock1
A. rpoB marker
Acidovorax
Sphingomonas
V. paradoxus
Sphingobacterium
Delftia
S. liquefaciens
S. maltophilia
Acinetobacter
E. mundtii
Achromobacter
P. protegens
P. cholororaphis
A. faecalis
Paenibacillus
P. putida
O. anthropi
B. diminuta
P. luminescens
X. nematophila
Expected
Illumina_Mock4
Expected
Illumina_Mock5

## Slide 2
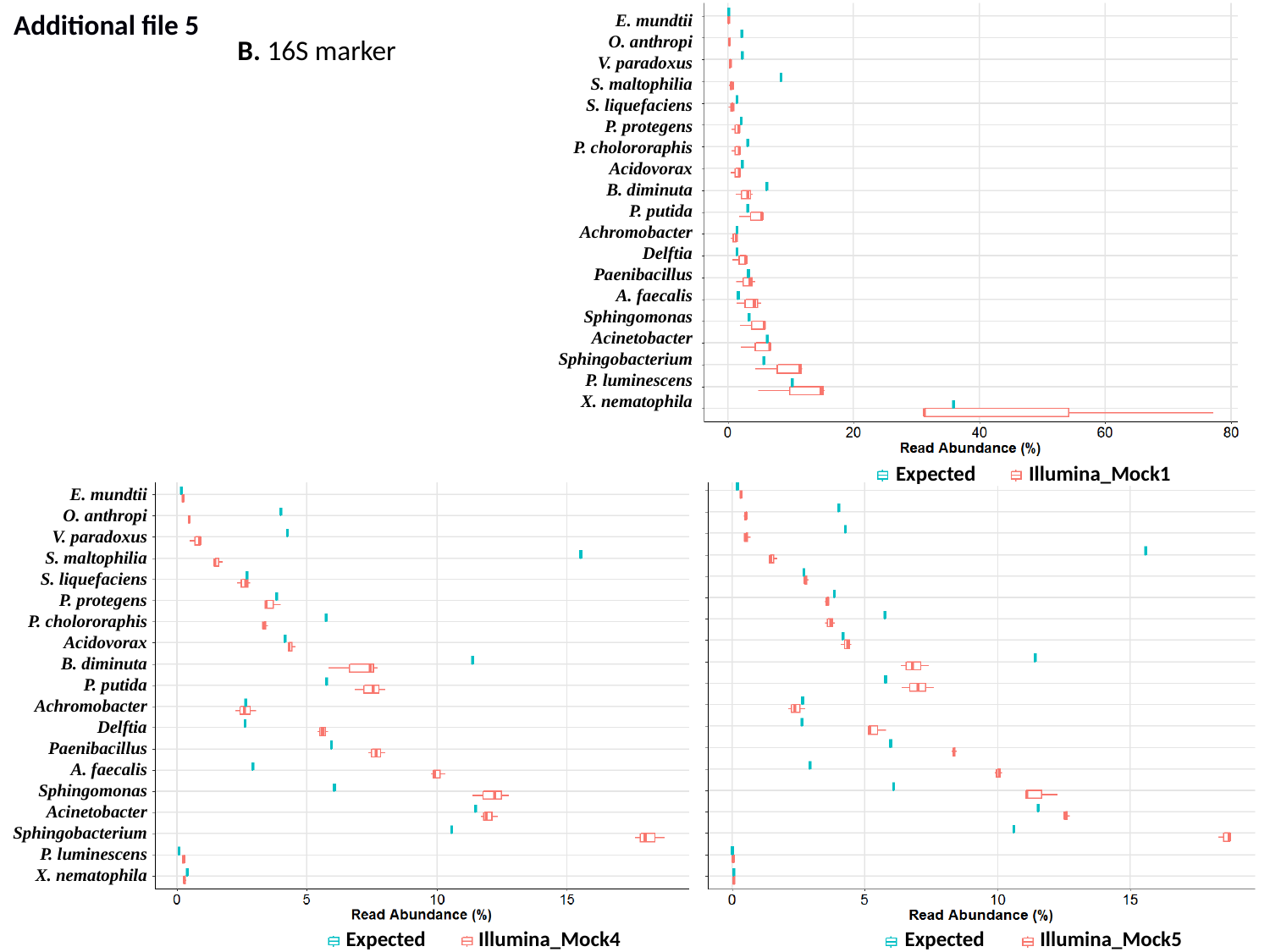

Additional file 5
E. mundtii
O. anthropi
V. paradoxus
S. maltophilia
S. liquefaciens
P. protegens
P. cholororaphis
Acidovorax
B. diminuta
P. putida
Achromobacter
Delftia
Paenibacillus
A. faecalis
Sphingomonas
Acinetobacter
Sphingobacterium
P. luminescens
X. nematophila
B. 16S marker
Expected
Illumina_Mock1
E. mundtii
O. anthropi
V. paradoxus
S. maltophilia
S. liquefaciens
P. protegens
P. cholororaphis
Acidovorax
B. diminuta
P. putida
Achromobacter
Delftia
Paenibacillus
A. faecalis
Sphingomonas
Acinetobacter
Sphingobacterium
P. luminescens
X. nematophila
Expected
Illumina_Mock4
Expected
Illumina_Mock5
