# Supplementary material for: rpoB, a promising marker for analyzing the diversity of bacterial communities by amplicon sequencing
Source: BMC Microbiol. 2019 Jul 29;19:171. doi: 10.1186/s12866-019-1546-z (PMC6664775; doi:10.1186/s12866-019-1546-z)
Supplement: Supplementary file 7 — Comparison of the bacterial communities associated with nematode samples (Steinernema glaseri SK39) and extraction control samples. (PPTX 80 kb) [file 12866_2019_1546_MOESM7_ESM.pptx]

## Slide 1
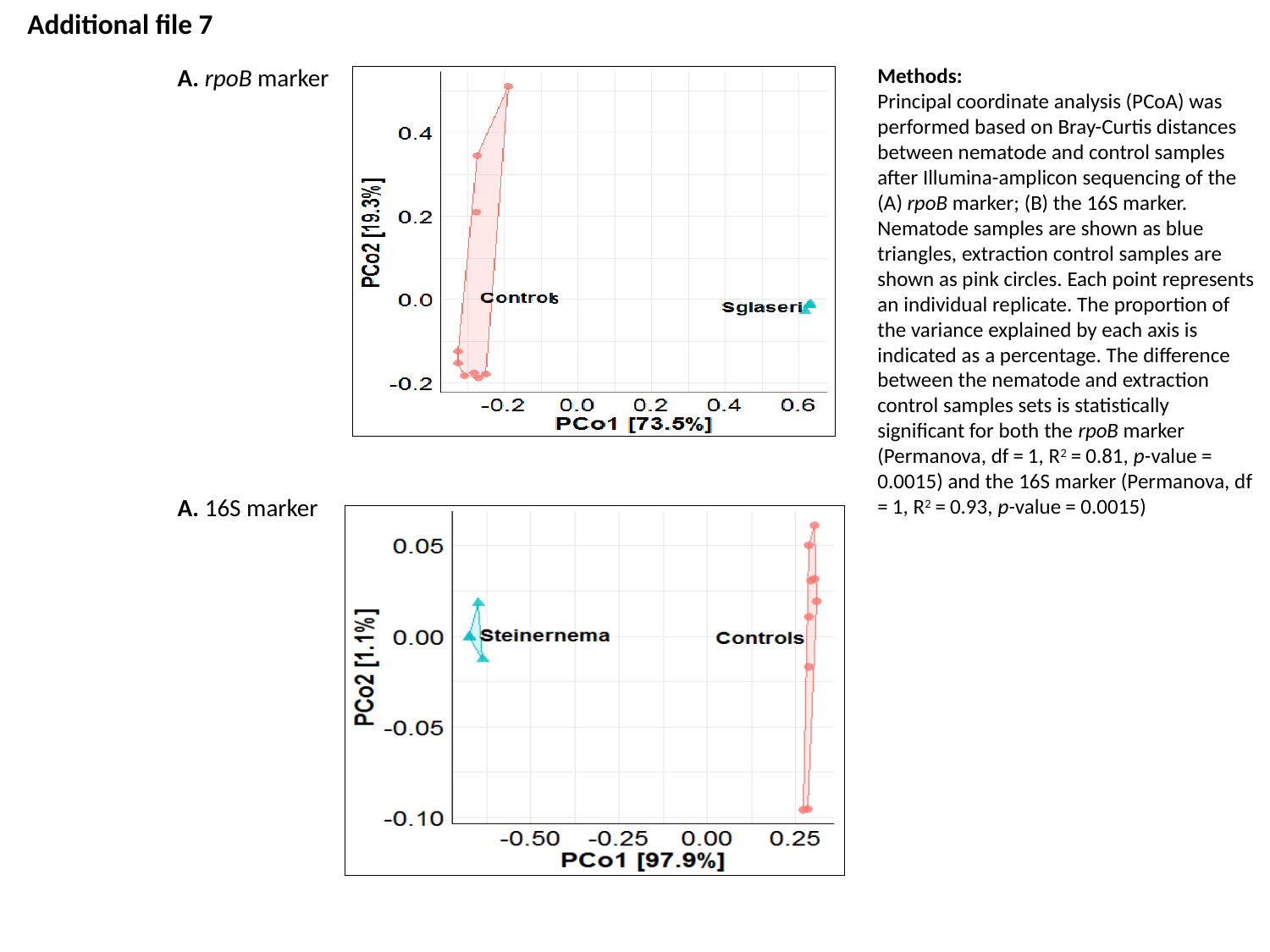

Additional file 7
A. rpoB marker
Methods:
Principal coordinate analysis (PCoA) was performed based on Bray-Curtis distances between nematode and control samples after Illumina-amplicon sequencing of the (A) rpoB marker; (B) the 16S marker. Nematode samples are shown as blue triangles, extraction control samples are shown as pink circles. Each point represents an individual replicate. The proportion of the variance explained by each axis is indicated as a percentage. The difference between the nematode and extraction control samples sets is statistically significant for both the rpoB marker (Permanova, df = 1, R2 = 0.81, p-value = 0.0015) and the 16S marker (Permanova, df = 1, R2 = 0.93, p-value = 0.0015)
s
A. 16S marker
