# Supplementary material for: rpoB, a promising marker for analyzing the diversity of bacterial communities by amplicon sequencing
Source: BMC Microbiol. 2019 Jul 29;19:171. doi: 10.1186/s12866-019-1546-z (PMC6664775; doi:10.1186/s12866-019-1546-z)
Supplement: Supplementary file 9 — List of biological materials used in the experimental study (DOCX 39 kb) [file 12866_2019_1546_MOESM9_ESM.docx]

**Additional file 9**

**A. List of the bacterial strain isolates used to generate the mock communities**

| **Species** | **Strain** | **Source of isolation** | **Reference** |
| --- | --- | --- | --- |
| *Xenorhabdus nematophila* | F1 | Symbiont of *S. carpocapsae* SK27 (hanging drop isolation technique) | [[1](#_ENREF_1), [2](#_ENREF_2)] |
| *Photorhabdus luminescens* | TT01 | Symbiont of *H. bacteriophora TT01(hanging drop isolation technique)* | [[3](#_ENREF_3), [4](#_ENREF_4)] |
| *Pseudomonas protegens* | SW4 | *G. mellonella cadaver after IJ infestation with S. weiseri* 583 | This study |
| *Pseudomonas chlororaphis* | SK39 ApoA | *G. mellonella* cadaver after IJ infestation with *S. glaseri* SK39 | This study |
| *Pseudomonas putida* | SW5 | *G. mellonella* cadaver after IJ infestation with *S. weiseri* 583 | This study |
| *Stenotrophomonas maltophilia* | B163 | *G. mellonella* cadaver after IJ infestation with *S. carpocapsae SK27* | This study |
| *Achromobacter sp.* | SK39 ApoC | *G. mellonella* cadaver after IJ infestation with *S. glaseri* SK39 | This study |
| *Alcaligenes faecalis* | SC | Isolated after the crushing of *S. carpocapsae* SK27 IJs | This study |
| *Ochrobactrum anthropi* | SW2 | *G. mellonella* cadaver after IJ infestation with *S. weiseri* 583 | This study |
| *Acinetobacter* | FR211 A | *G. mellonella* cadaver after IJ infestation with *S. affine* | This study |
| *Serratia liquefaciens* | TUR03-2-2 | Isolated from *S. weiserii* 583 (hanging drop isolation technique) | This study |
| *Delftia sp.* | MW8A-4 | Isolated from storage medium of a batch of *S. tanzaniensis* IJs | This study |
| *Variovorax sp.* | BS 3-2-1 | Isolated after the crushing of  *H. bacteriophora* BS 3.2 IJs | This study |
| *Acidovorax sp.* | 173 | Isolated after crushing of  *S. xueshanense* Dequen IJs | This study |
| *Sphingomonas sp.* | BG | Isolated after the crushing of *H. bacteriophora* CS3-2 IJs | This study |
| *Brevundimonas sp.* | TCH07-Apo2-4 | Isolated from *S. weiserii 583* by the hanging drop technique | This study |
| *Sphingobacterium sp.* | FR211C | *G. mellonella* cadaver after IJ infestation with *S. affine* | This study |
| *Paenibacillus sp.* | SK72-2 | *G. mellonella* cadaver after IJ infestation with *S. glaseri* SK72 | This study |
| *Enterococcus mundtii* | SG4 | *G. mellonella* cadaver after IJ infestation with *S. glaseri* SK39 | This study |

**B. List of the primers used in this study**

| **Primer label** | **Sequence (5’-3’)** | **Utilization** | **Reference** |
| --- | --- | --- | --- |
| 16S_F | GAAGAGTTTGATCATGGCTC | 16S rRNA gene Sanger sequencing | [[5](#_ENREF_5)] |
| 16S_R | AAGGAGGTGATCCAGCCGCA |  |  |
| Univ_rpoB_F_deg | **GGYTWYGAAGTNCGHGACGTDCA** | *rpoB* gene fragment Sanger sequencing | This study |
| Univ_rpoB_R_deg | **TGACGYTGCATGTTBGMRCCCATMA** |  |  |
|  |  |  |  |
| F343 | CTTTCCCTACACGACGCTCTTCCGATCTTA**CGGRAGGCAGCAG** | V3V4 Illumina-amplicon sequencing | [[6](#_ENREF_6)] |
| R784 | GGAGTTCAGACGTGTGCTCTTCCGATCTTA**CCAGGGTATCTAATCCT** |  |  |
|  |  |  |  |
| Univ_rpoB_F_deg | CTTTCCCTACACGACGCTCTTCCGATCT**GGYTWYGAAGTNCGHGACGTDCA** | *rpoB* Illumina-amplicon sequencing | This study |
| Univ_rpoB_R_deg | GGAGTTCAGACGTGTGCTCTTCCGATCT**TGACGYTGCATGTTBGMRCCCATMA** |  |  |

1. Akhurst RJ, Boemare NE. A numerical taxonomic study of the genus *Xenorhabdus* (*Enterobacteriaceae*) and proposed elevation of the subspecies of *X. nematophilus* to species. J Gen Microbiol. 1988;134:1835-45.

2. Sicard M, Brugirard-Ricaud K, Pages S, Lanois A, Boemare NE, Brehelin M, et al. Stages of infection during the tripartite interaction between *Xenorhabdus nematophila*, its nematode vector, and insect hosts. Appl Environ Microbiol. 2004;70:6473-80.

3. Boemare NE, Akhurst RJ, Mourant RG. DNA Relatedness between *Xenorhabdus* Spp (*Enterobacteriaceae*), Symbiotic Bacteria of Entomopathogenic Nematodes, and a Proposal to Transfer *Xenorhabdus* *luminescens* to a New Genus, *Photorhabdus* Gen-Nov. Int J Syst Evol Bacteriol. 1993;43:249-55.

4. Duchaud E, Rusniok C, Frangeul L, Buchrieser C, Givaudan A, Taourit S, et al. The genome sequence of the entomopathogenic bacterium *Photorhabdus luminescens*. Nat Biotechnol. 2003;21:1307-13.

5. Tailliez P, Pages S, Ginibre N, Boemare N. New insight into diversity in the genus *Xenorhabdus*, including the description of ten novel species. Int J Syst Evol Microbiol. 2006;56:2805-18.

6. Klindworth A, Pruesse E, Schweer T, Peplies J, Quast C, Horn M, et al. Evaluation of general 16S ribosomal RNA gene PCR primers for classical and next-generation sequencing-based diversity studies. Nucleic Acids Res. 2013;41:e1.
